# Silencing of Cholinergic Basal Forebrain Neurons Using Archaerhodopsin Prolongs Slow-Wave Sleep in Mice

**DOI:** 10.1371/journal.pone.0130130

**Published:** 2015-07-07

**Authors:** Yu-Feng Shi, Yong Han, Yun-Ting Su, Jun-Hua Yang, Yan-Qin Yu

**Affiliations:** Department of Neurobiology and Physiology, Key Laboratory of Medical Neurobiology of the Ministry of Health of China, Key Laboratory of Neurobiology, Zhejiang University School of Medicine, Hangzhou, Zhejiang, China; Imperial College London, UNITED KINGDOM

## Abstract

The basal forebrain (BF) plays a crucial role in cortical activation. Our previous study showed that activation of cholinergic BF neurons alone is sufficient to suppress slow-wave sleep (SWS) and promote wakefulness and rapid-eye-movement (REM) sleep. However, the exact role of silencing cholinergic BF neurons in the sleep-wake cycle remains unclear. We inhibitied the cholinergic BF neurons genetically targeted with archaerhodopsin (Arch) with yellow light to clarify the role of cholinergic BF neurons in the sleep-wake cycle. Bilateral inactivation of cholinergic BF neurons genetically targeted with archaerhodopsin prolonged SWS and decreased the probability of awakening from SWS in mice. However, silencing these neurons changed neither the duration of wakefulness or REM sleep, nor the probability of transitions to other sleep-wake episodes from wakefulness or REM sleep. Furthermore, silencing these neurons for 6 h within the inactive or active period increased the duration of SWS at the expense of the duration of wakefulness, as well as increasing the number of prolonged SWS episodes (120-240 s). The lost wakefulness was compensated by a delayed increase of wakefulness, so the total duration of SWS and wakefulness during 24 h was kept stable. Our results indicate that the main effect of these neurons is to terminate SWS, whereas wakefulness or REM sleep may be determined by co-operation of the cholinergic BF neurons with other arousal-sleep control systems.

## Introduction

The basal forebrain (BF) consists of a group of structures, including the nucleus basalis, substantia innominata, diagonal band of Broca, and medial septal nuclei, which provide the major cholinergic innervation of the cerebral cortex [1–3]. The BF plays an important role in rapid-eye-movement (REM) sleep and wakefulness through its wide projections to the cerebral cortex and innervation from the brainstem and other nuclei involved in the sleep-wake cycle [4, 5]. Acetylcholine and cholinergic neurons are known to play a critical role in stimulating cortical activation in the waking state as well as in REM sleep [5–7]. Cholinergic neuronal activity and acetylcholine release reach maximal levels during REM sleep [8–10], and are maintained at high levels during wakefulness. Identified cholinergic BF neurons discharge at their highest rates during active wakefulness and REM sleep [9], indicating that they are involved in promoting cortical activation during these states. However, controversial results have been reported regarding the specific role of cholinergic BF neurons in the sleep-wake cycle. Microinjection of neurotensin into the BF was proposed to selectively stimulate cholinergic neurons, and induced cortical activation with decreased slow-wave sleep (SWS), enhanced REM sleep, and increased quiet waking state [11]. On the other hand, microinjection of carbachol (a cholinergic agonist) into the BF of cats and dogs reduces SWS and increases wakefulness in the subsequent 4 h [12, 13]. Also, microinjection of carbachol into the BF reduces the ability to elicit REM sleep by simultaneous injection of carbachol into the pons [13]. These results are inconsistent and carbachol may affect both cholinergic and non-cholinergic neurons [14]. Furthermore, using immunotoxin 192 IgG-saporin to lesion cholinergic BF neurons in rats has been reported to significantly inhibit cortical activation with reduced high-frequency electroencephalographic (EEG) activity, but does not affect sleeping and waking behavior [4]. In addition, recent studies have shown only minor EEG changes after extensive cholinergic-specific lesions in the BF [15, 16].

Recently-developed optogenetic tools [17] provide a valuable means of stimulating or inhibiting activity in genetically-targeted neuronal populations with high spatial and temporal precision [18, 19]. We demonstrated that activation of cholinergic BF neurons genetically-targeted with channelrhodopsin 2 is sufficient to suppress SWS and promote wakefulness and REM sleep [20]. But it was still not clear if cholinergic BF neurons are necessary for maintaining arousal. To identify a conclusive, causal role of the cholinergic BF system in natural sleep-waking events, we selectively silenced these neurons in freely-moving ChAT-Arch-GFP (choline acetyltransferase-archaerhodopsin-green fluorescent protein) transgenic mice (referred to below as ChAT-Arch mice).

## Materials and Methods

### Animals and genotyping

All procedures were approved by the Zhejiang University Animal Research Advisory Committee (Hangzhou, China). All surgery was performed under sodium pentobarbital anesthesia, and all efforts were made to minimize suffering. Experiments were performed on adult wild-type (C57) and ChAT-Arch-GFP transgenic mice (2–3 months old, 25–30 g, male). The animals were housed under a 12/12-h light/dark cycle (starting at 07:00) in cages with up to 4 other animals before the implants and alone after the implants. After experiments, the mice in all groups were sacrificed by intraperitoneal administration of pentobarbital sodium overdose.

To selectively inactivate cholinergic neurons, we crossed ChAT-Cre mice (Jackson Laboratories, *B6;129S6-Chat<tm1(cre)Lowl>/J*, stock number 006410) with *loxP*-flanked Arch-GFP reporter mice (Jackson Laboratories, *129S-Gt(ROSA)26Sor<tm35*.*1(CAG-AOP3/GFP)Hze>/J*, stock number 012735). We used Ai35D mice (ss-Arch-GFP-ER2), which conditionally express an improved Arch-GFP driven by Cre-mediated removal of the STOP cassette, to cross with ChAT-Cre mice. The modified version of Arch was chosen because it produces larger conductance changes, likely owing to its slower deactivation kinetics [21]. When bred to ChAT-Cre mice, the resulting *ChAT-Cre/Ai35D* heterozygotic offspring (ChAT-Arch mice) had the STOP cassette deleted in the cholinergic neurons, resulting in expression of the Arch-GFP fusion protein. Cre-negative, Ai35D heterozygotic littermates were used as controls in all experiments. Four control mice and 10 ChAT-Arch mice were used in this study. Anesthesia was induced with pentobarbital sodium (100 mg/kg, i.p.).

For genotype identification by PCR, the offspring (2–3 weeks old) were gently hold and the tail was fixed with fingers so the tip of tail tissue was easily cut off by surgical scissor and collected into the Eppendorf tube, followed by digestion with 100 μl NaOH (25 mM) in Eppendorf tubes heated in a 95°C water-bath for 1 h and then neutralized by 300 μl Tris-HCl (40 mM). After the PCR procedure was completed, the Ai35D and Cre gene bands were revealed by 2% agarose gel electrophoresis.

### Brain slice preparation

Adult ChAT-Arch mice were sacrificed by pentobarbital sodium overdose and decapitated. The whole brain was quickly dissected into ice-cold oxygenated artificial cerebrospinal fluid (aCSF) containing (in mM): 130 NaCl, 5 KCl, 2.4 CaCl_2_, 1.3 MgSO_4_, 1.25 KH_2_PO_4_, 10 glucose, and 20 NaHCO_3_, pH 7.4 with NaOH, bubbled with a mixture of 95% O_2_ and 5% CO_2_. Then the brain was cut coronally into 300-μm slices on a microtome (VTA-1000S; Leica). Slices containing the BF were transferred to an incubation chamber filled with aCSF and incubated for at least 1 h at room temperature (24–26°C).

### *In vitro* electrophysiological recordings

At room temperature, the slices were transferred to a recording chamber on a fluorescence microscope stage (BX51WI; Olympus) and maintained immersed and continuously superfused with aCSF at 4–5 ml/min. Patch electrodes were pulled on a micropipette puller (P-97; Sutter Instruments) from borosilicate capillaries (GC150-10; Harvard Apparatus). The pipettes (5–12 MΩ) contained the following solution (in mM): 97.5 K-gluconate, 32.5 KCl, 1 MgCl_2_.6H_2_O, 40 HEPES, 0.5 Na-GTP, 2 Mg-ATP, and 0.5 EGTA, pH 7.4. Recordings were made with a MutiClamp 700B amplifier (Molecular Devices). Neurons that expressed GFP fluorescence were identified as cholinergic and were subjected to current-clamp recordings. Traces were low-pass filtered at 10 kHz and digitized at 10 kHz (MICRO3 1401, Cambridge Electronic Design). Data were acquired and analyzed using Spike2 7.04 software (Cambridge Electronic Design).

For inactivation of cholinergic BF neurons, the optical fiber (numerical aperture 0.37 and core radius 200 μm) was coupled to a 589-nm solid-state laser diode (Shanghai Laser & Optics Century Co., Ltd). The fiber was passed through a stainless-steel tube (inner diameter 250 μm, outer diameter 480 μm) and bonded to the tube with glue (AC-001, Aron alpha). The tip of the fiber was trimmed and polished, submerged in aCSF, and placed above the stimulating site. Yellow light pulse-trains (20 Hz/20 ms for 30 s and 60 s) or continuous yellow illumination (30 s and 60 s) were delivered. The power of the laser (0.5–1.5 mW) was measured by a power meter (PM10, Conherent) before experiments.

### Surgical implantation

ChAT-Arch and control mice respectively received unilateral and bilateral surgical implantation of a custom-made EEG and electromyographic (EMG) unit placed on the rear of the skull. Animals were anesthetized with pentobarbital sodium (100 mg/kg, i.p.) and mounted on a small-animal stereotaxic frame (Stoelting Corp.). Before the EEG/EMG eletrode was implanted 4 skull screw-holes placed on the left/right frontal cortices (AP, +2 mm; ML, 1 mm) and left/right occipital cortex (AP, -3 mm; ML, 2 mm) were drilled, into which screws fit tightly and were driven through the skull to the surface of the dura. EEG signals were recorded from right frontal screw and the reference electrode was left occipital screw, and the other two stainless-steel screws were used for anchorage to the skull. Two stainless-steel wires were placed into the neck muscles for EMG recording. Then the animals were implanted with the guide cannulas (RWD Life Science) posterior to the EEG/EMG eletrode implantation. The cannula was placed above the right or bilateral BF [anteroposterior (AP), -0.7 mm; mediolateral (ML), 1.6 mm; dorsoventral, 4.2 mm]. Both the EEG/EMG electrodes and cannula were fixed to skull with dental cement. After experiment the optical fiber tract and the location of photostimulation were examed.

After the surgical procedures, the animals were allowed to recover in individual cages for at least 7 days. Each animal was transferred to a recording chamber and connected to an EEG/EMG head-stage and the optical fiber. The data cable was connected to a slip-ring device (CFS-22, Biotex) so that mice could freely move in their cages without tangling the cable. An optical commutator (FRJ_FC-FC, Doric Lenses) was used to release torsion in the optical fiber caused by the animal’s movements. The animals were habituated for 3 days before EEG and EMG recording.

### EEG and EMG recording

The EEG and EMG signals from the implanted electrodes were amplified, filtered (EEG, 0.5–100 Hz; EMG, 20–200 Hz), and recorded using a preamplifier (Model 1700 Differential AC Amplifier, A-M Systems) and amplified 1000× with a filter frequency range from 0.1 Hz to 500 Hz for both EEG and EMG. Signals were then sampled at 200 Hz with an ML795 PowerLab/4SP data acquisition system (AD Instruments) and stored on a hard disk for off-line analysis.

### *In vivo* optogenetic inactivation

*In vivo* inactivation experiments were conducted bilaterally on the BF. Two optical fibers were inserted into the guide cannulas that had been implanted one day before the experiments (at 13:00). The other end of the fiber was attached to a rotating optical commutator (FRJ_FC-FC, Doric Lenses) to permit free movement of the mouse. The 30-s continuous yellow illumination was programmed using the PG-4000A (Cygnus Technology) pulse stimulator. Evidence suggests that low levels of light intensity (e.g. 0.35 mW/mm^2^) are sufficient for Arch activation [22]. The power of yellow light we applied was 0.8 mW at the tip of the fiber, which produced >0.35 mW/mm^2^ up to 0.7 mm directly away from the fiber tip. For acute inactivation, experiments were carried out from 13:00 to 17:00 in the inactive period and 22:00 to 02:00 in the active period. Thirty-second continuous yellow illumination was applied 12 s after the onset of a stable wakefulness, SWS, or REM sleep event, as detected by real-time online EEG/EMG analysis. The probabilities of light-induced wake-to-SWS, SWS-to-awake/REM sleep and REM-awake transitions during the 30-s continuous yellow illumination in inactive period or active period were measured by offline scoring of the EEG/EMG recordings and expressed as a percentage of state changes. The duration of SWS and REM sleep episodes was measured at baseline and with 30-s inactivation during the inactive and active periods. The baseline was determined by analyzing the duration of individual episodes during the same time on the day before inactivation.

For chronic inactivation, we used programmed inactivation (continuous for 60 s, once every 2 min). To compare the effects of different chronic inactivation across a 24-h cycle, we divided the 24 h into an inactive period (07:00 to 19:00) and an active period (19:00 to 07:00). Animals were stimulated for 6 h from 13:00 to 19:00 or 19:00 to 01:00. After recording, the duration of wakefulness, SWS, and REM sleep per hour and the percentage of wakefulness, SWS, and REM sleep in 24 h in control and ChAT-Arch mice were quantified by offline scoring. These animals were not subjected to stimulation or sleep recordings again for at least 7 days.

### Sleep-wake state classification

Sleep state was scored with sleep analysis software (SleepSign). All scoring was automatic on the basis of the signature of the EEG and EMG waveforms in 4-s epochs. We defined wakefulness as desynchronized EEG and heightened tonic EMG activity with phasic bursts; SWS as synchronized, high-amplitude, low-frequency (0.5–4 Hz) EEG and greatly reduced EMG activity compared with wakefulness with no phasic bursts; and REM sleep as having a pronounced theta rhythm (4–10 Hz) and a flat EMG (atonia). All classifications of states assigned by SleepSign were examined visually and corrected manually, if necessary.

### EEG power spectral analyses

The power spectral density of the digitally-filtered EEG signals were analyzed by fast Fourier transformation using the NeuroExplorer (Nex Technology) software in a 0–35 Hz window with 0.38 Hz resolution. The duration of individual EEG used for power spectral density analysis was 30 s before and after beginning of photoinhibition. To normalize the data, we used the relative EEG power represented by the ratio of the power spectral density in the different frequency ranges to the average value of total power in the same epoch. So the changes of the EEG power spectral density during a 30-s baseline and 30-s photoinhibition among SWS, REM sleep, and wakefulness were analyzed. The baseline was defined by calculating the 30-s EEG power spectral density at about the same time on the day before photoinhibition.

### Immunohistochemistry

For immunohistochemistry, adult mice were deeply anesthetized with pentobarbital sodium (100 mg/kg, i.p.) and transcardially perfused with cold normal saline followed by 4% paraformaldehyde in 0.1 M PBS. The brain was post-fixed for 4 h and then cryoprotected in 30% sucrose for at least 24 h. After embedding in optimal cutting temperature compound, the brain was sectioned coronally at 30 μm on a freezing microtome (CM 1950; Leica). Sections were rinsed with 0.5% Triton-X in 0.1 M PBS and blocked with 10% normal bovine serum for 1 h. For ChAT immunohistochemistry, sections were incubated with primary antibody (rabbit anti-ChAT, 1:200, Millipore AB143) in 0.1 M PBS for 12 h at 4°C. Sections were then rinsed and incubated with Cy3-conjugated goat anti-rabbit IgG antibody (1:1000, Jackson ImmunoResearch; 2 h at room temperature). The analysis of immunostained neurons was performed immediately to avoid fading of the fluorescence. Two sections were bilaterally analysed in each mouse, five mice were used. The area of substantia innominata, basal nucleus, ventral pallidum, magnocellular preoptic nucleus and nucleus of the horizontal limb of the diagonal band in BF were analysed.

### Statistical analysis

Data are presented as mean ± s.e.m. Student’s *t*-test and one or two-way ANOVA followed by Tukey post-hoc test was used for statistical analysis. In all cases, *p* <0.05 was taken as the level of significance.

## Results

### Identification of ChAT-Arch-GFP transgenic mice

**Fig 1:**
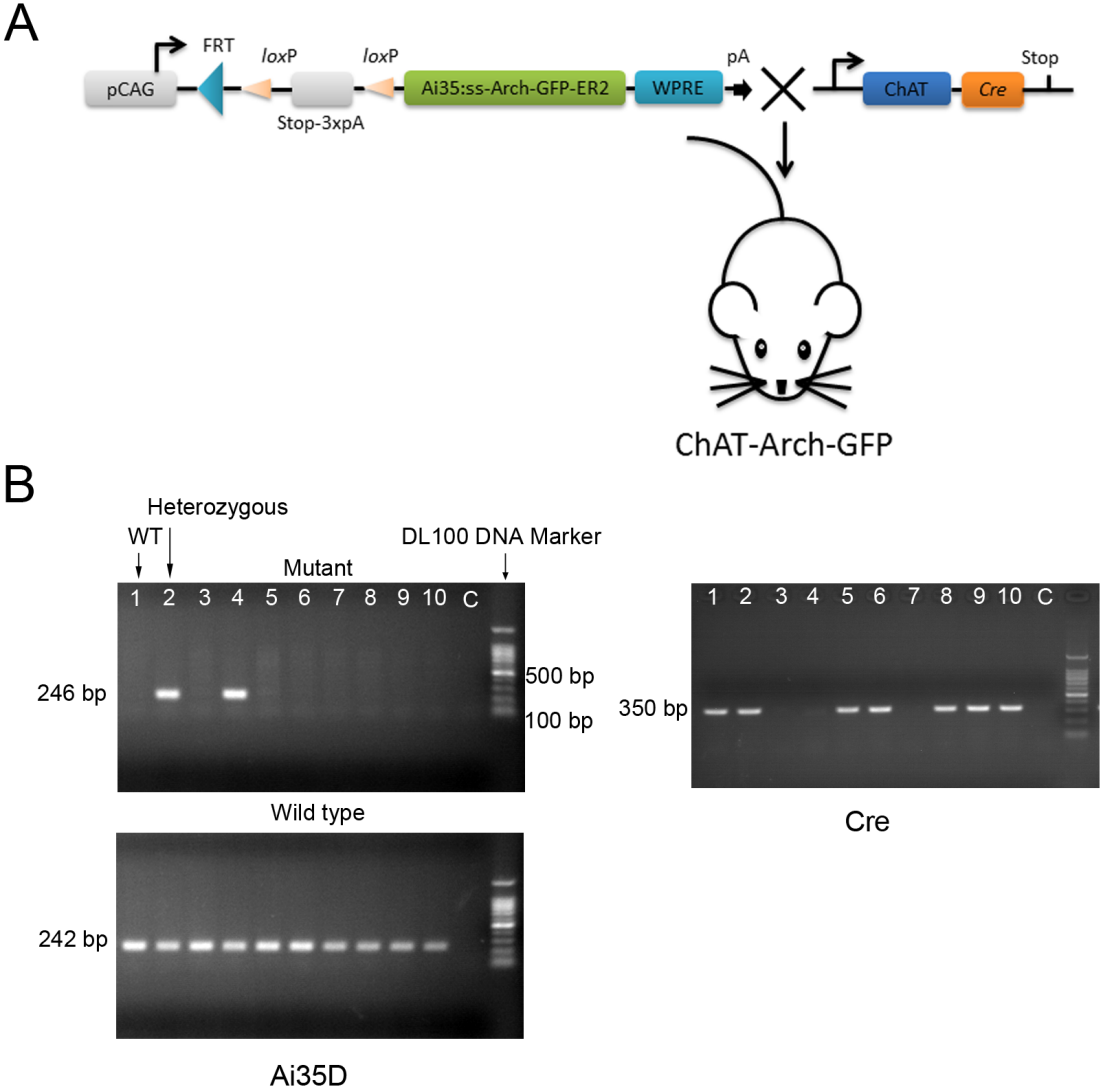
Recombination of Ai35D and ChAT-Cre mice. **(A**) Diagram of heterozygous mice with conditional expression of Arch-GFP after recombination of the Stop-loxP gene and ChAT-Cre recombinase. (**B**) PCR analysis. Ai35D heterozygous mice had one 242-bp and one 246-bp band, while WT mice had only one 242-bp band. The positive Cre band was at 350-bp.

To selectively express Arch in cholinergic BF neurons, we obtained *ChAT-Cre/Ai35D* heterozygotes (ChAT-Arch mice) by breeding Ai35D mice to ChAT-Cre mice as described in the Methods (Fig 1A). The Ai35D heterozygotes were viable and fertile. They were identified by PCR and agarose gel electrophoresis with one 242-bp band and one 246-bp band (Fig 1B). Meanwhile, we also tested for the Cre gene in the ChAT-Arch mice. The positive Cre band after electrophoresis was 350 bp (Fig 1B)

#### Genetic targeting of cholinergic neurons in the BF

**Fig 2:**
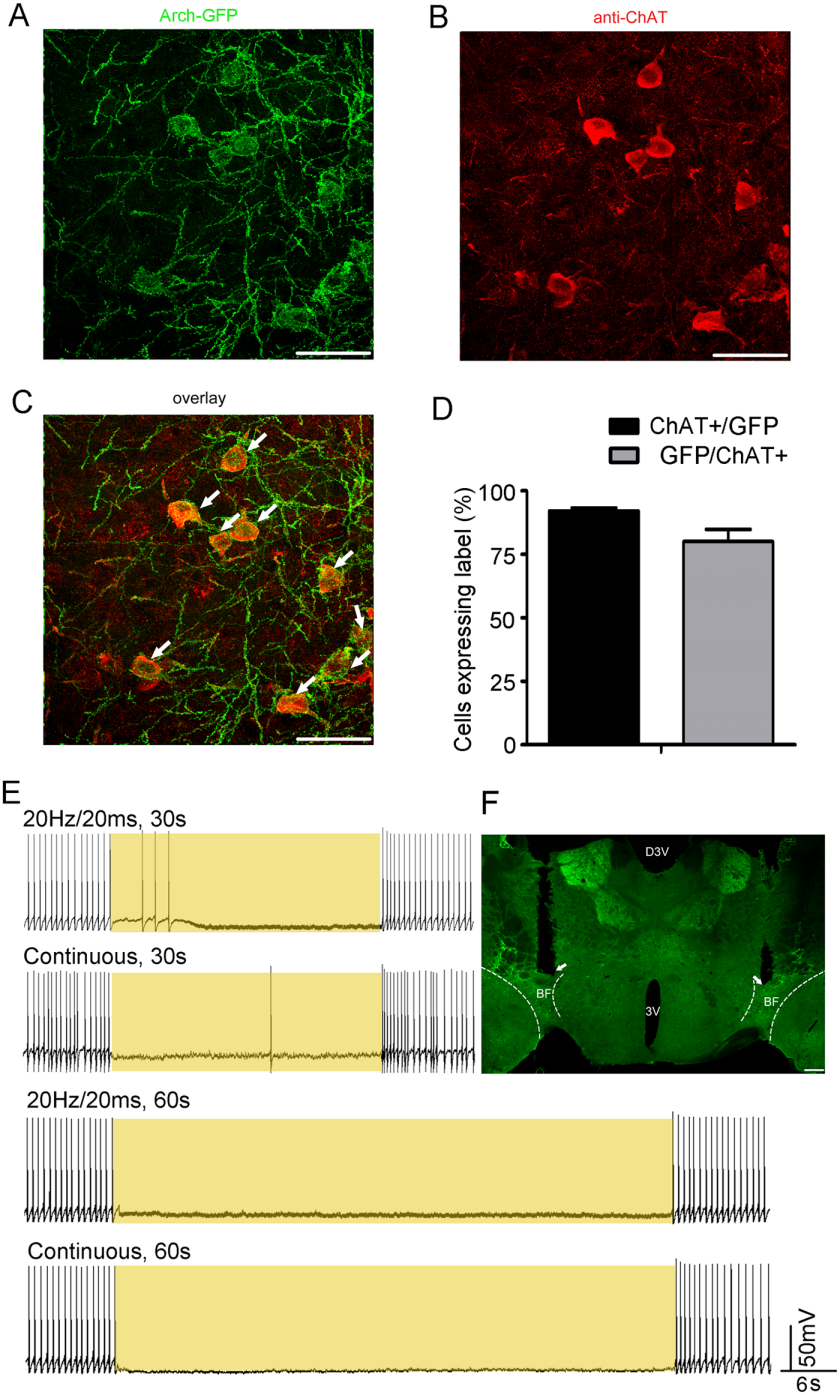
Specific and efficient expression of Arch-GFP in cholinergic BF neurons of ChAT-Arch mice. (**A**–**C)** Representative photomicrographs of the BF depicting Arch-GFP expression (**A**, green), ChAT immunoreactivity (**B**, red), and overlaid images (**C**) from a ChAT-Arch mouse. Scale bars, 50 μm. (**D)** Statistics of the total co-expression of GFP and ChAT immunofluorescence from ChAT-Arch mice (n = 5) in 30-μm sections from the rostral-to-caudal ends of the BF (anteroposterior, -0.4 to -0.8). Cell counts are represented as mean ± s.e.m. **(E)** Yellow light inhibits the activity of Arch-expressing cholinergic BF neurons. Action potentials recorded under current-clamp from a neuron expressing Arch-GFP in a BF slice in response to 20 Hz yellow light pulse trains (20 ms per pulse) or continuous yellow light for 30 s (upper trace). Action potentials recorded under current-clamp from a neuron expressing Arch-GFP in a BF slice in response to 20 Hz yellow light pulse trains (20 ms per pulse) or continuous yellow light for 60 s (lower trace). (**F**) Representative photomicrographs of Arch-GFP expression in BF from a ChAT-Arch mouse. White arrow indicated a fiberoptic lesion track. 3V, 3rd ventricle; D3V, dorsal 3rd ventricle. Scale bars, 200 μm.

To confirm that the expression of Arch-GFP was restricted to cholinergic neurons, we carried out immunohistochemical reactions for ChAT in the ChAT-Arch mice. Merged images revealed that Arch-GFP occurred mostly in cholinergic neurons (Fig 2A–2C). Out of 381 neurons in the BF that expressed ChAT (n = 5 mice), 80.1 ± 4.8% also expressed GFP, showing that the Arch-GFP expression was efficient. Conversely, 92.1 ± 1.2% of GFP-positive cells in the BF also expressed ChAT (Fig 2D), which showed that the genetic targeting in the BF was highly specific. The results showed that Arch-GFP was selectively expressed with high efficiently in the cholinergic BF neurons of ChAT-Arch mice.

### Yellow light inactivates Arch-expressing cholinergic BF neurons

To test the function of Arch expressed in cholinergic BF neurons in the ChAT-Arch mice, whole-cell recordings were performed in brain slices. As reported previously, cholinergic BF neurons have large somata (≥23 μm) and are characterized electrophysiologically by the presence of a short-lasting rectification [23]. Yellow light-induced inactivation of cholinergic BF neurons was confirmed using whole-cell recording. Thirty seconds of yellow light (20 Hz/20 ms and continuous) almost completely inhibited spontaneous firing in Arch-expressing cholinergic BF neurons (Fig 2E). Next, to determine the duration of the effect, yellow light was continuously applied for 60 s. Whole-cell recording revealed that yellow light immediately inhibited spontaneous firing (Fig 2E). When illumination was terminated, firing immediately returned to the basal level. Three cells were recorded in one mouse. These results strongly suggested that illumination of the BF inhibits the activity of cholinergic neurons and that Arch expression reliably inactivates these neurons.

### Changes in sleep-wake episodes by inactivation of cholinergic BF neurons

**Fig 3:**
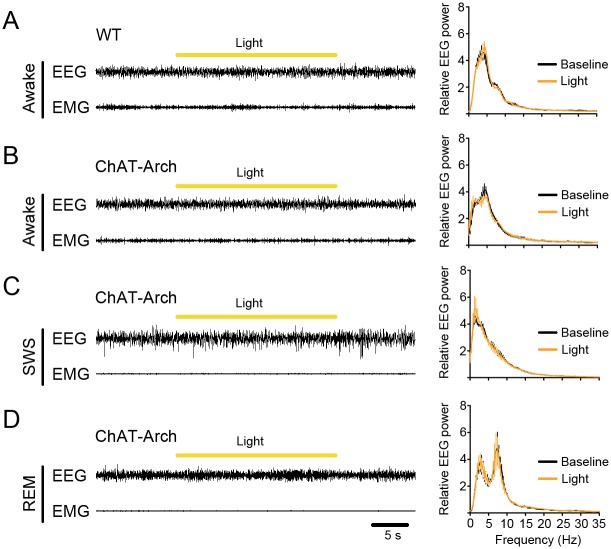
Photoinhibition of cholinergic BF neurons during wakefulness, SWS, and REM sleep in the inactive period. (**A**–**D)** Left panel, representative EEG and EMG recordings showing that photoinhibition (yellow bars, continuous yellow light for 30 s) applied during waking (**B**), SWS (**C**), or REM sleep (**D**) in a ChAT-Arch mouse and during waking (**A**) in a wild-type mouse. Right panel, relative cortical EEG power (0.38-Hz binned frequencies, 30-s duration) at baseline (black trace) and after the onset of photostimulation (yellow trace) in ChAT-Arch mice. n = 3 mice, 10 stimulations per mouse in **A**–**D**. *p* >0.05, two-tailed Student’s t test between baseline and ChAT-Arch mice with bilateral inactivation of cholinergic BF neurons.

To investigate the regulation of sleep-wake behavior by cholinergic BF neurons, we performed 30-s *in vivo* illumination of the BF with yellow light in freely-moving ChAT-Arch mice during wakefulness, SWS, and REM sleep (Fig 3A–3D). We checked the effect of the light (continuous, 30 s) delivered separately during the inactive period (13:00 to 17:00) and the active period (22:00 to 02:00), and found that illumination did not change the relative EEG power spectrum compared with baseline (*p* >0.05; Fig 3).

**Fig 4:**
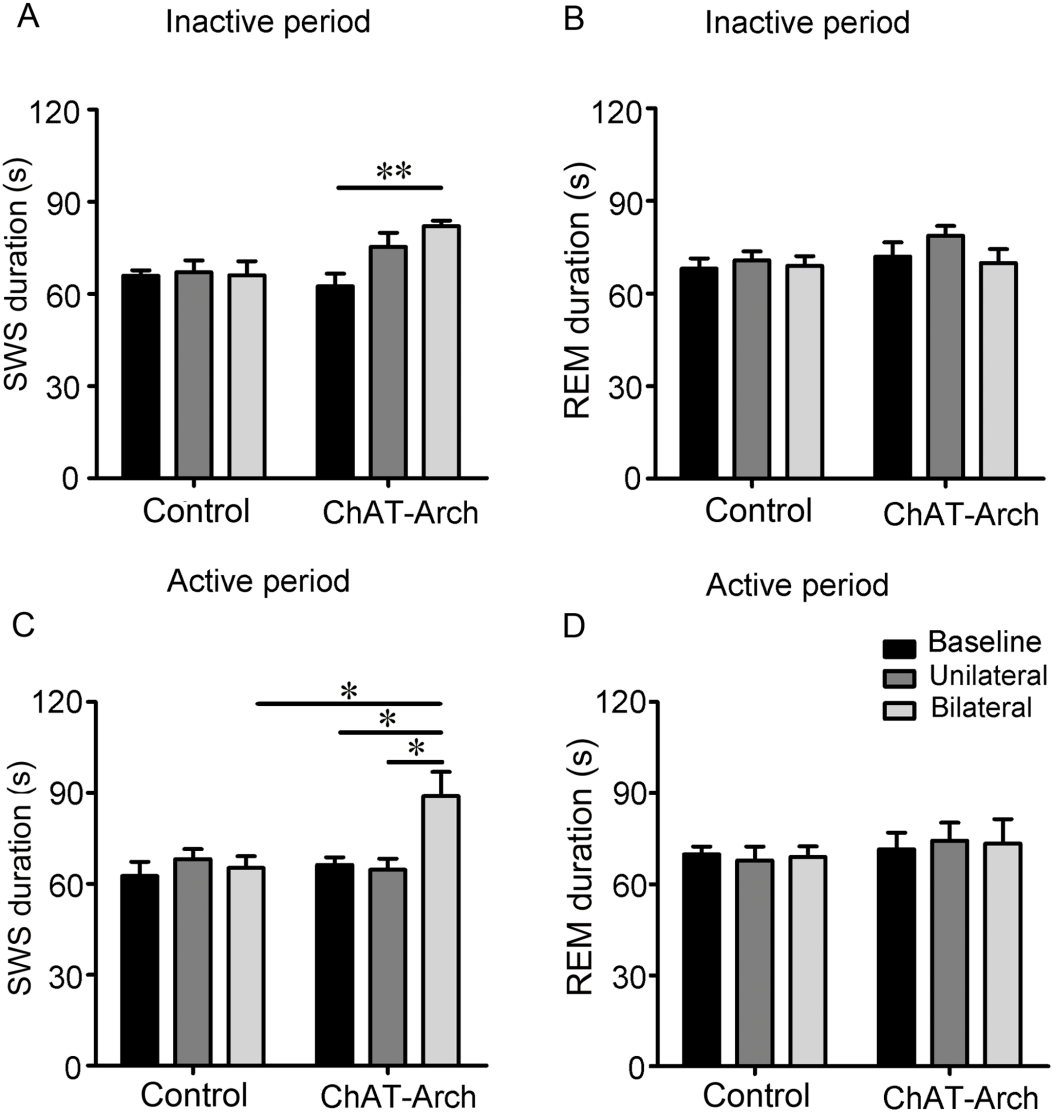
Silencing of cholinergic BF neurons prolongs SWS duration but does not affect REM sleep. (**A**, **C)** Duration of individual SWS episodes at baseline and 30-s unilateral or bilateral continuous inactivation during the inactive period (**A**) and active period (**C**). (**B**, **D)** Duration of individual REM sleep episodes at baseline and 30-s unilateral or bilateral continuous inactivation during the inactive period (**B**) and active period (**D**). The experiments were performed from 13:00 to 17:00 during the inactive period and from 22:00 to 02:00 during the active period. The baseline was determined by analyzing the duration of individual SWS or REM sleep episodes about the same time on the day before inactivation. n = 5 mice, *p <0.05, **p <0.01, two-way ANOVA between stimulation condition and animal type followed by Tukey’s *post-hoc* test.

During the inactive period, bilateral inactivation of cholinergic BF neurons in the ChAT-Arch mice during SWS extended its duration from a baseline of 62.5 ± 4.2 s to 82.1 ± 1.8 s (*p* <0.01; Fig 4A). But the duration of SWS with 30-s unilateral inactivation was 75.3 ± 4.5 s and not significantly different from baseline (Fig 4A). There was no difference in the duration of SWS in the control mice (67.1 ± 3.9 s, unilateral; 66.1 ± 4.6 s, bilateral) compared with baseline (66.0 ± 1.8 s).

In the active period, the SWS duration also increased from 66.2 ± 2.5 s to 88.9 ± 8.0 s with 30-s continuous bilateral inactivation (*p* <0.05; Fig 4C). There was a significant duration extension of SWS between bilateral inactivation and unilateral inactivation in the ChAT-Arch mice or bilateral lluminalion in control mice (*p* <0.05; Fig 4C). But the duration of SWS with 30-s unilateral inactivation was 64.6 ± 3.7 s, which was not significantly different from baseline in the active period (Fig 4C). There was no difference in the duration of SWS in control mice (68.2 ± 3.3 s, unilateral; 65.2 ± 3.9 s, bilateral) compared with baseline (62.6 ± 4.7 s).

During the inactive or active period, the duration of REM sleep was not affected by 30-s continuous unilateral or bilateral inactivation of cholinergic BF neurons (Fig 4B and 4D). In the inactive period, the duration of REM sleep in ChAT-Arch mice was from 72.0 ± 4.6 s to 78.7 ± 3.1 s (unilateral, *p* >0.05) and 70.0 ± 4.5 s (bilateral, *p* >0.05) with 30-s continuous inactivation (Fig 4B), and in the active period was from 71.5 ± 5.4 s to 74.3 ± 5.9 s (unilateral, *p* >0.05) and 73.4 ± 8.0 s (bilateral, *p* >0.05) (Fig 4D).

### Silencing cholinergic BF neurons reduces the transition from SWS

**Fig 5:**
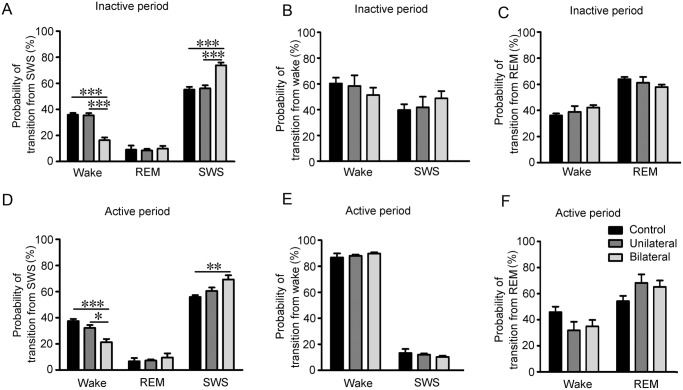
Silencing of cholinergic BF neurons reduces transitions from SWS but does not affect transitions from wakefulness and REM sleep. (**A**, **D)** Percentage probability of transition to wakefulness or REM sleep or maintained SWS from SWS within a single 30-s unilateral or bilateral continuous inactivation in the inactive period (**A**) and the active period (**D**). (**B**, **E)** Percentage probability of transition to SWS or maintained wakefulness from wakefulness within a single 30-s unilateral or bilateral continuous inactivation in the inactive period (**B**) and the active period (**E**). (**C**, **F)** Percentage probability of transition to wakefulness or maintained REM sleep from REM sleep within a single 30-s unilateral or bilateral continuous inactivation in the inactive period (**C**) and the active period (**F**). n = 6 mice, *p <0.05, **p <0.01, ***p <0.001, one-way ANOVA for unilateral or bilateral continuous inactivation compared with control followed by Tukey *post-hoc* test.

Then we analyzed the probabilities of light-induced awake-to-SWS, SWS-to-awake/REM sleep, and REM-awake transitions in the inactive and active periods. During the inactive period, the ChAT-Arch mice had a 16.4 ± 2.2% probability of an SWS-to-awake transition during 30-s bilateral continuous inactivation (Fig 5A), however, in control mice and ChAT-Arch mice with unilateral inactivation, the probability was 35.8 ± 1.4% (*p* <0.001, compared with bilateral inactivation) and 35.4 ± 1.7% (*p* <0.001, compared with bilateral inactivation; Fig 5A). Accordingly, there was a significantly increased probability of remaining in SWS during 30-s bilateral inactivation in the ChAT-Arch mice (73.8 ± 2.1%) compared with control mice (55.1 ± 2.1%, *p* <0.001) and ChAT-Arch mice with unilateral inactivation (56.1 ± 2.4%, *p* <0.001, Fig 5A). But there was no difference of the transition from SWS-to-REM sleep in the ChAT-Arch mice (8.5 ± 1.1%, unilateral and 9.9 ± 2.0%, bilateral) compared with control (9.0 ± 3.1%) with 30-s inactivation of cholinergic BF neurons (*p* >0.05; Fig 5A).

During the active period, the ChAT-Arch mice had a 21.2 ± 2.5% probability of SWS-to-awake transition during 30-s bilateral continuous inactivation (Fig 5D), however, in control mice and ChAT-Arch mice with unilateral inactivation of cholinergic BF neurons, the probability was 37.4 ± 1.6% (*p* <0.001, compared with bilateral inactivation) and 32.2 ± 2.2% (*p* <0.05, compared with bilateral inactivation; Fig 5D). Similarly, there was a significantly increased probability of remaining in SWS during 30-s bilateral inactivation in the ChAT-Arch mice (69.3 ± 3.2%) compared with control mice (55.8 ± 1.5%, *p* <0.01; Fig 5D) and ChAT-Arch mice with unilateral inactivation (60.6 ± 2.6%). But there was also no difference of the transition from SWS-to-REM sleep in the ChAT-Arch mice (7.2 ± 0.8%, unilateral; 9.5 ± 3.2%, bilateral) compared with control mice (6.7 ± 2.4%) during 30-s inactivation of cholinergic BF neurons in the active period (*p* >0.05; Fig 5D).

In contrast to the results described above, 30-s bilateral continuous inactivation of cholinergic BF neurons in awake, freely-moving ChAT-Arch mice had no effect on the awake-to-SWS transition during the inactive or active periods (Fig 5B and 5E). In the inactive period, awake-to-SWS transition in ChAT-Arch mice was 41.7 ± 8.3% (unilateral, *p* >0.05) and 48.7 ± 5.7% (bilateral, *p* >0.05) compared with control (39.7 ± 4.5%) during 30-s continuous inactivation of cholinergic BF neurons (Fig 5B). In the active period, the awake-to-SWS transition in ChAT-Arch mice was 12.0 ± 0.9% (unilateral, *p* >0.05) and 10.3 ± 0.9% (bilateral, *p* >0.05) compared with control (13.3 ± 3.1%) during 30-s continuous inactivation (Fig 5E). Likewise, when the ChAT-Arch mice were in REM sleep, 30-s bilateral continuous inactivation did not change the REM-to-awake transition during the inactive or active period (Fig 5C and 5F). In the inactive period, REM-to-awake transition in ChAT-Arch mice was 38.9 ± 4.5% (unilateral, *p* >0.05) and 42.2 ± 1.9% (bilateral, *p* >0.05) compared with control (36.0 ± 1.6%) during 30-s continuous inactivation (Fig 5C). In the active period, REM-to-awake transition in ChAT-Arch mice was 31.8 ± 6.6% (unilateral, *p* >0.05) and 34.9 ± 4.9% (bilateral, *p* >0.05) compared with control (45.8 ± 4.2%) during 30-s continuous inactivation of cholinergic BF neurons (Fig 5F).

These results suggested that silencing of cholinergic BF neurons has little effect on the initiation and maintenance of wakefulness or REM sleep, so these neurons may not be necessary for the maintenance of wakefulness and REM sleep.

### Long-term inactivation of cholinergic BF neurons in ChAT-Arch mice in the light/dark period

To investigate whether continuous inactivation of cholinergic BF neurons had long-term effects on sleep architecture, we analyzed the amount of wakefulness, SWS, and REM sleep per hour in the 24 h during and following inactivation.

**Fig 6:**
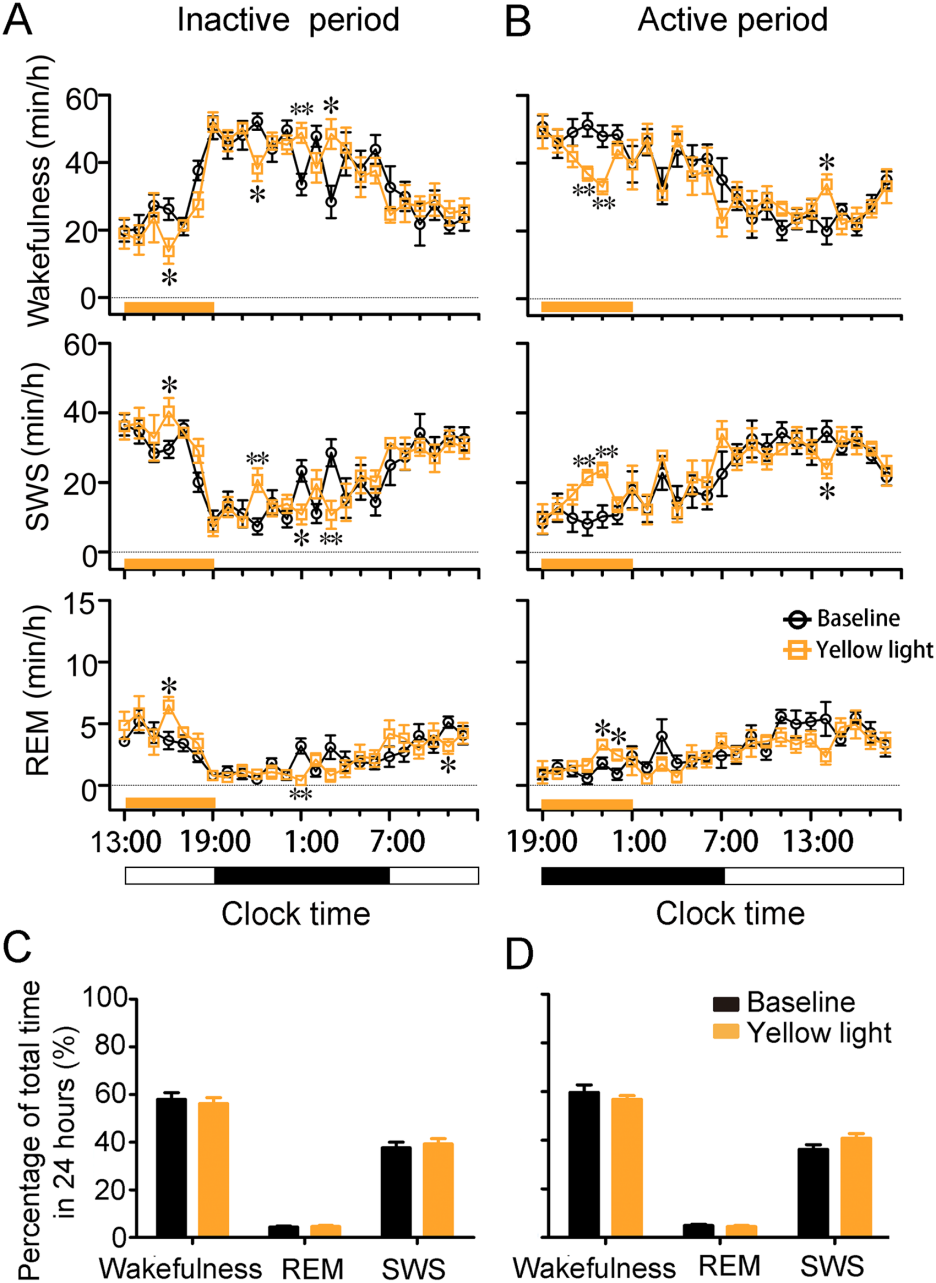
Six-hour inactivation of cholinergic BF neurons during the inactive or active period. **(A**, **B)** Time the ChAT-Arch mice spent in wakefulness, SWS, and REM sleep during 24 h with 6-h inactivation (continuous, 60 s, once every 2 min) in the inactive period (13:00–19:00) (**A**) and the active period (19:00–01:00) (**B**). Each symbol represents the hourly mean time in each stage. The baseline was determined by analyzing the hourly mean time in each stage on the day before inactivation. **(C, D)** Percentage of total times the ChAT-Arch mice spent in wakefulness, SWS, and REM sleep during 24 h with and without light inactivation. n = 6 mice, *p <0.05, **p <0.01, two-tailed Student’s t test between baseline and with light inactivation.

Six hours of inactivation (13:00 to 19:00; continuous, 60 s, once every 2 min) during the inactive (light) period in the ChAT-Arch mice increased the amount of SWS and REM sleep and decreased the amount of wakefulness during the 6 h (*p* <0.05; Fig 6A). In the first 3 h of inactivation, the amount of wakefulness, SWS, and REM sleep was not affected, apparently showing that the photoinhibition was not specific for these phenomena. During the fourth hour (16:00–17:00) the amount of wakefulness was decreased from the baseline of 26.6 ± 3.2% to 13.7 ± 4.5% (*p* <0.05; Fig 6A), while the amount of SWS and REM sleep were increased from a baseline of 29.6 ± 2.5% to 40.4 ± 3.9% (*p* <0.05; Fig 6A) and from a baseline of 3.7 ± 0.7% to 5.9 ± 0.6% (*p* <0.05; Fig 6A), respectively. During the subsequent 18 h, this long-term inactivation caused increased wakefulness (*p* <0.05; Fig 6A) and reduced SWS (*p* <0.05; Fig 6A) and REM sleep (*p* <0.01; Fig 6A) compared with baseline. However, the lost wakefulness was compensated by a delayed increase, thus the total duration of the SWS, wakefulness, and REM sleep during 24 h remained stable (Fig 6C). The same experiments performed during the active (dark) period (19:00 to 1:00) generated similar results (Fig 6B and 6D). The above results demonstrated that 6 h of inactivation of cholinergic BF neurons did not change the 24-h sleep architecture and the circadian rhythm, and sleep homeostasis was maintained in the normal range by compensatory mechanisms.

**Fig 7:**
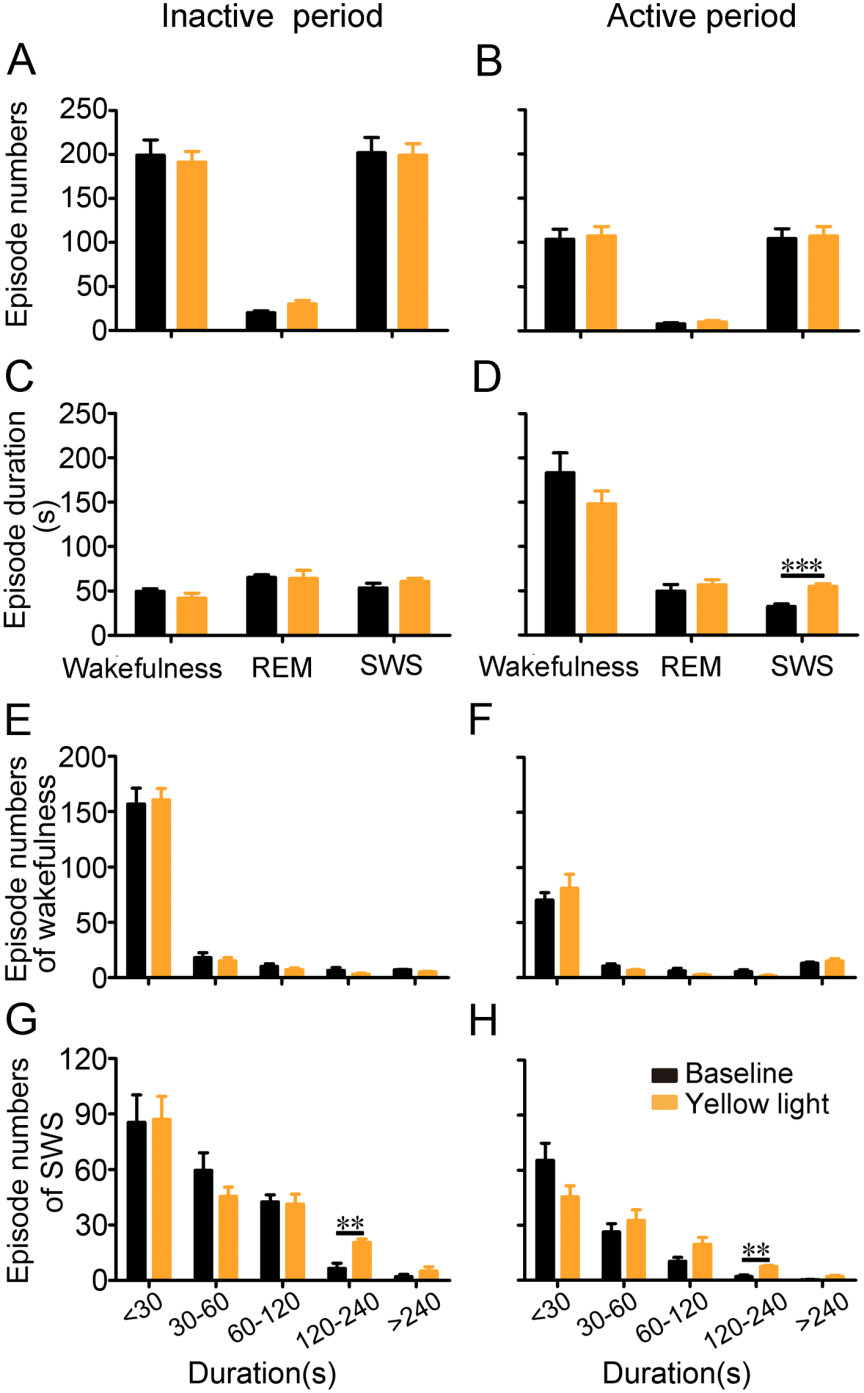
SWS episode duration increases during 6-h inactivation of cholinergic BF neurons during the active period. **(A, B)** Numbers of episodes of wakefulness, SWS, and REM sleep during 6 h with and without light inactivation. n = 5 ChAT-Arch mice. **(C**, **D)** Durations of episodes of wakefulness, SWS, and REM sleep during 6 h with and without light inactivation. n = 5 ChAT-Arch mice. **(E, F)** Numbers of episodes of wakefulness during 6 h with and without light inactivation. n = 5 ChAT-Arch mice. **(G, H)** Numbers of episodes of SWS during 6 h with and without light inactivation. n = 5 ChAT-Arch mice. **p <0.01, ***p <0.001, two-tailed Student’s t test between baseline and with light inactivation.

Next, we examined the number of episodes during inactivation and found that this number was not affected by 6 h of inactivation during the active or the inactive period (Fig 7A and 7B). It is interesting that the duration of SWS episodes increased with 6 h of inactivation in the active period rather than in the inactive period (*p* <0.001; Fig 7C and 7D). When we determined the distribution of episodes of wakefulness and SWS during 6 h of inactivation in active or inactive period, the inactivation increased the number of prolonged SWS episodes (120–240 s) (*p* <0.01; Fig 7G and 7H), whereas the number of wakefulness episodes was not affected (Fig 7E and 7F). These results showed that 6 h of inactivation of cholinergic BF neurons during the inactive and active periods caused increased SWS and decreased wakefulness by increasing the duration and number of SWS episodes.

## Discussion

In the present study, bilateral silencing of the cholinergic BF neurons by light prolonged SWS duration by reducing SWS-to-awake transitions, indicating the importance of cholinergic activity in normal sleep processing. It neither changed the duration of wakefulness and REM sleep, nor the transitions to other sleep-wake states from wakefulness or REM sleep. This result indicated that cholinergic BF neurons are sufficient to promote wakefulness or REM sleep but play a non-exclusive role in this process. Combined with our previous study [20], these results show that cholinergic BF neurons play two roles: to terminate SWS and to promote wakefulness or REM sleep; they promote wakefulness or REM sleep by terminating SWS.

Previous work has shown that selective lesioning of cholinergic BF neurons with 192IgG-Sapori transiently increases SWS by 13%, predominantly during the dark period [15]. Deletion of these neurons (~95%) produces a decrease in theta activity during REM sleep [24], but does not change the overall daily levels of wakefulness, SWS, or REM sleep [25]. Another report showed a blunted increase in recovery of the SWS delta wave [26] in a transgenic mouse model of Alzheimer’s disease with loss of cholinergic BF neurons [27]. It has been hypothesized that the cholinergic BF, histaminergic tuberomammillary nucleus, and noradrenergic locus ceruleus neurons are key links in the circuit regulating arousal [28]. But rats with lesions of all 3 of these neuronal phenotypes have overall daily levels of wakefulness not significantly different from saline-treated rats (Blanco-Centurion et al., 2007). Although these pharmacological lesion experiments had limited specificity, and they caused more damage than the optogenetic method, their results are in agreement with our findings that the cholinergic BF neurons are not necessary for maintaining arousal or for REM sleep.

The interpretation of our results of temporarily silencing the cholinergic BF neurons and the above results of lesion or loss of these neurons might be because the other arousal groups such as orexinergic neurons in the lateral hypothalamus [18], the histaminergic neurons in the tuberomammillary nucleus [29], the noradrenergic locus ceruleus neurons [30], serotoninergic neurons in the dorsal raphe nuclei [31], cholinergic neurons in the pedunculopontine and lateral tegmental nuclei [32, 33], and other cell-types in the BF [34] compensate and stabilize the sleep-wake network. Our results suggest that the cholinergic BF, along with other arousal nuclei, jointly modulate arousal and REM sleep, but they are not essentially linked to the brain circuitry responsible for daily levels of wakefulness and REM sleep, as traditionally hypothesized.

Our previous study showed that the activation of cholinergic BF neurons facilitates the transition from SWS to wakefulness or REM sleep, but does not change the direction of the transition [20], whereas the Arch-mediated inactivation of these neurons had the opposite effects on SWS in the present study. Thus, cholinergic BF neurons potentially play a powerful role in the rapid modulation of SWS by interrupting it for another sleep-wake episode. Compared to the previous studies showing that the cholinergic BF neurons play an important role in promoting cortical arousal, it is possible that the main effect of activating these neurons is to terminate SWS in order to permit a transition to the next episode, whereas the type of transition (to wakefulness or to REM sleep) may be determined by other neuronal systems or by co-operation of the cholinergic BF neurons with other arousal-sleep control systems [6, 35]. Silencing of the cholinergic BF neurons in the present study indicated that they play a more important role in SWS regulation than promoting wakefulness or REM sleep. The BF has an efferent projection to the lateral preoptic area in which the ventral part is active in SWS [36, 37]. The substantia innominata of the BF is reported to project to the SWS-promoting parvicellular area of the reticular nucleus (also called the parafacial zone) [38, 39]. So in future studies, it will be important to clarify the anatomical and functional connections between cholinergic BF neurons and SWS-on nuclei such as the GABAergic neurons in the ventrolateral preoptic area [40] and the parafacial zone [38].

In addition to the immediate sleep-wake transitions induced by short trains of light pulses, we found that sustained inactivation (6 h) of cholinergic BF neurons had a long-term effect on sleep and wakefulness, characterized by prolonged SWS and REM sleep with a decreased duration of wakefulness that occurred during light inactivation. An increased duration of REM sleep could follow the increased SWS episode during the 6-h light inactivation. Interestingly, a delayed rebound of the lost wakefulness occurred several hours after 6-h light inactivation, no matter whether the stimulation was applied during the active or the inactive period. Sleep is regulated by two basic physiological processes, the diurnal rhythm and sleep homeostasis [41]. The diurnal rhythm did not change in the sustained light-inhibited mice, since these animals maintained a stable sleep architecture and sleep-wake rhythm compared with baseline (Fig 6). Thus, these changes may be due to sleep homeostatic regulation because sleep-enhancement during the 6-h inactivation decreased the SWS during the post-stimulation period due to decreased homeostatic sleep pressure. Since the inactivation of cholinergic BF neurons prolonged SWS duration, acetylcholine in the brain may also serve as a therapeutic target for the treatment of insomnia.
