# Correction: Silencing of Cholinergic Basal Forebrain Neurons Using Archaerhodopsin Prolongs Slow-Wave Sleep in Mice

**DOI:** 10.1371/journal.pone.0134421

**Published:** 2015-07-28

**Authors:** The PLOS ONE Staff

There are errors in the Funding section. The correct funding information is as follows: This work was supported by the National High Technology Research and Development Program of China (863 Program) (2015AA020512) and the National Natural Science Foundation of China (81371458). The publisher apologizes for the errors.
